# Controlled human malaria infection—Maker and breaker of dogma

**DOI:** 10.1371/journal.pmed.1003591

**Published:** 2021-04-26

**Authors:** Jean-Marc Chavatte, Georges Snounou

**Affiliations:** 1 Malaria Reference Centre–National Public Health Laboratory, National Centre for Infectious Diseases, Singapore; 2 Commissariat à l’Energie Atomique et aux Energies Alternatives-Université Paris Sud 11-INSERM U1184, Immunology of Viral Infections and Autoimmune Diseases (IMVA-HB), Infectious Disease Models and Innovative Therapies (IDMIT) Department, Institut de Biologie François Jacob (IBFJ), Direction de la Recherche Fondamentale (DRF), Fontenay-aux-Roses, France

## Abstract

Jean-Marc Chavatte and Georges Snounou discuss research involving controlled malaria infections.

Experimental human infections have contributed significantly to knowledge about infectious diseases [1]. In a recent report in *PLOS Medicine*, Rebecca Watts and colleagues followed this tradition by describing controlled human infection (CHI) with artemisinin-resistant *Plasmodium falciparum* [2]. Of the short list of pathogens still utilized in CHIs [1], malaria is exceptional because much of our knowledge of its natural course and its parasites’ (*Plasmodium*) biology derives from an extensive series of CHIs that stretches over a century. The trigger to investigate malaria through routine CHIs was provided by Julius Wagner-Jauregg. Convinced of a beneficial effect of fever on psychoses, he infected patients suffering from neurosyphilis with *Plasmodium vivax* and recorded unprecedented full recoveries or partial remissions in most [3]. Consequently, malariotherapy centers flourished worldwide, attracting malariologists who derived unique insights into the nature of malaria from these CHIs [4]. CHIs became so important for malaria studies that when malariotherapy was superseded by antibiotics, malariologists initiated ethically sanctioned volunteer programs.

While malariotherapy relied primarily on *P*. *vivax*, *P*. *falciparum* was used mainly in the United States of America on African American patients who were refractory to *P*. *vivax* and from the 1940s, on volunteers of all ethnic backgrounds. From the mid-1970s, the majority of CHIs were initiated by *P*. *falciparum* sporozoites, principally to serve in the development of a vaccine against the preerythrocytic forms. These investigations have a minimal clinical risk as the volunteers are treated as soon as blood stages are detectable (a parasite (P) burden ≈ 10 P/μL), a level in initial infection rarely preceded by any clinical sign. By contrast, for blood stage–induced *P*. *falciparum* infections that were mainly conducted to evaluate drug efficacy, curative drug treatment was to be initiated as parasite levels neared 1,000 P/μL, but in some volunteers, these exceeded 10,000 P/μL because of the unpredictable and often fulminating multiplication of *P*. *falciparum*. Thus, by the mid-1980s, blood stage inoculations were abandoned on ethical grounds.

Allan Saul and his team at the Queensland Institute of Medical Research (QIMR) broke this moratorium by bringing together 3 elements needed to embark on CHI initiated by blood stage malaria parasites. First, they elaborated a strategy to constitute a stock of infected red blood cells (RBCs) uncontaminated by other pathogens suitable to serve as an inoculum. Second, they devised a sensitive quantitative method of parasite detection (≈10 P/mL) allowing the monitoring of parasitemia over a sufficient number of replication cycles prior to the appearance of any clinical signs. Finally, a protocol ensuring the safety of the volunteers was elaborated, and ethical approval was secured. Thus, a stock of cryopreserved RBCs infected by *P*. *falciparum* 3D7 was obtained and validated [5], making it possible to conduct CHIs using a standard inoculum. Then, a limited number of studies aiming at testing the efficacy of various vaccine formulations [6] served to further develop, refine, and establish the induced blood stage model (IBSM). From the early 2010s, the team led by James McCarthy at the QIMR Berghofer Research Institute (Queensland, Australia) turned the IBSM into an important strand of malariological research, both scientific and translational.

McCarthy’s team showcased the IBSM in a first study in which the parasite reduction rates of atovaquone–proguanil (Malarone) and artemether–lumefantrine (Coartem, an early artemisinin-based combination therapy or ACT) were determined [7] and found to agree with observations made in numerous clinical trials. In the new study [2], they have revisited the clearance rate of artesunate, another artemisinin derivative, but here used alone as a single dose against 2 *P*. *falciparum* cloned lines: 3D7, a sensitive line, and a resistant line Cam3.II^R539T^ (K13^R539T^ in [2]) derived from an isolate collected in Cambodia [8]. In a pilot study, the authors inoculated 2 volunteers with K13^R539T^ and established that this infection was safe and well tolerated. This provided the green light for the main study that compared the parasite clearance half-lives of these 2 lines by artesunate: that of the sensitive 3D7 line (3.2 h in 9 volunteers) was found to be half that of the resistant K13^R539T^ line (6.5 h in 13 volunteers). This comparative study is of significance for several reasons. The *P*. *falciparum* K13^R539T^ line is the first cloned artemisinin-resistant line that was banked and validated for use in CHI studies. Furthermore, this approach opens the potential to extend investigations to other parasite lines, to uncover the molecular mechanisms that underly artemisinin resistance, and also to investigate other biological processes of malaria parasites.

The emergence and spread of parasites resistant to artemisinin derivatives poses the direst threat to the malaria control and elimination roadmap. In the mid-2000s, robust indications of parasites with reduced sensitivity to ACTs were obtained from clinical field trials of artesunate in Cambodia, which showed a prolongation of the time to parasite clearance [9,10]. Unexpectedly, this was not accompanied by an increase in the in vitro–determined IC_50_ of artesunate [9,10]. Thereafter, the ring-stage survival assay that correlated with parasite clearance dynamics was developed [11], and the combination of these 2 parameters now defines resistance to artemisinin. This unorthodox feature initially led some in the malaria community to question whether this represents “true” resistance or even the beginning of it. Those still unconvinced favor resistance to the companion drug administered de facto by ACT treatment as an alternative interpretation of slower parasite clearance. This might well be true for some parasites, but both interpretations are not mutually exclusive. The study of Watts and colleagues [2] does provide a clear-cut demonstration that the delayed clearance phenomenon can be clearly attributed to exposure to artesunate alone, although it did not demonstrate that the particular mutation in the *Pf*kelch13 gene alone was responsible for the extended clearance half-life. It is highly likely that the initial reticence to view delayed clearance as an indication, or even a forerunner, of artemisinin resistance contributed to missing a window of opportunity to stop the spread of artemisinin-resistant *P*. *falciparum* in Cambodia. Artemisinin-resistant parasites have by now spread to other Southeast Asian countries [12].

From a historical perspective, it is gratifying to note the revival of experimental infections in humans and to appreciate their significant contributions to accelerating drug discovery and vaccine development. Their value, however, should not be limited to translational ends, because they offer a unique opportunity to define the breadth of the biological characteristics of other malaria parasite species of humans that are not amenable to in vitro investigations. McCarthy’s team has recently derived cryopreserved stocks of *P*. *vivax–*[13,14] and *Plasmodium malariae–*[15] infected RBCs for future CHIs. Observations from CHI are justifiably restricted by ethical considerations, yet they considerably advance our understanding of malaria.

## Abbreviations

ACT: artemisinin-based combination therapy
CHI: controlled human infection
IBSM: induced blood stage model
QIMR: Queensland Institute of Medical Research
P: parasite
RBC: red blood cell
